# Congenital afibrinogenemia: a case report of a spontaneous hepatic hematoma

**DOI:** 10.1097/MD.0000000000004150

**Published:** 2016-07-18

**Authors:** Stephanie Malaquin, Lionel Rebibo, Cyril Chivot, Louise Badoux, Yazine Mahjoub, Herve Dupont

**Affiliations:** a Surgical Intensive Care Unit; b Department of Digestive and Metabolic Surgery; c Department of Radiology, Amiens Picardy University Hospital; d INSERM U1088, University of Picardy Jules Verne, Amiens, France.

**Keywords:** congenital afibrinogenemia, hemorrhagic shock, spontaneous hepatic hematoma

## Abstract

**Introduction:**

Afibrinogenemia is a rare coagulation disorder. Clinical features of spontaneous bleeding, bleeding after minor trauma, or after surgery have been described as well as thrombo-embolic complications. In this article, we presented the case of a 19-year old female with congenital afibrinogenemia who was admitted with a spontaneous intrahepatic hematoma.

**Conclusions:**

Supportive treatment including transfusion and fibrinogen administration, associated with repeated packing surgeries and selective embolization, were successfully performed.

## 1 Introduction

Afibrinogenemia has an estimated prevalence of one for 1,000,000.^[1,2]^ It is an autosomal recessive disease that occurs as a result of mutations in 1 of the 3 fibrinogen genes: FGA, FGB, and FGG located on chromosom 4q.^[3]^ It is characterized by the complete absence or reduced amounts of immunoreactive fibrinogen as measured by antigenic and functional assays (less than 0.1 g/L). The most common clinical symptoms are mucocutaneous, soft-tissue, joint and genito-urinary spontaneous bleeding, traumatic, or surgical bleeding.^[4]^ Excessive menstrual bleeding in women is also reported.^[5–7]^ Thrombosis, poor wound healing, and splenic rupture are rarely reported.

## 2 Case report

A 19-year-old female patient with a history of congenital afibrinogenemia. According to the French law, in case of retrospective study, the ethical approval was not necessary.

She was admitted to the emergency department of a local hospital with acute abdominal pain and nausea. Her genetic fibrinogen mutation was allele 1: FGG IVS2–3c>G, allele 2: FGG IVS2–3c>G. She was being treated monthly with fibrinogen concentrates, tranexamic acid (during menstrual periods), and oral contraception. She had suffered from massive bleeding 7 years ago, diagnosed as hemoperitoneum complicating an ovarian cyst rupture.

**Figure 1:**
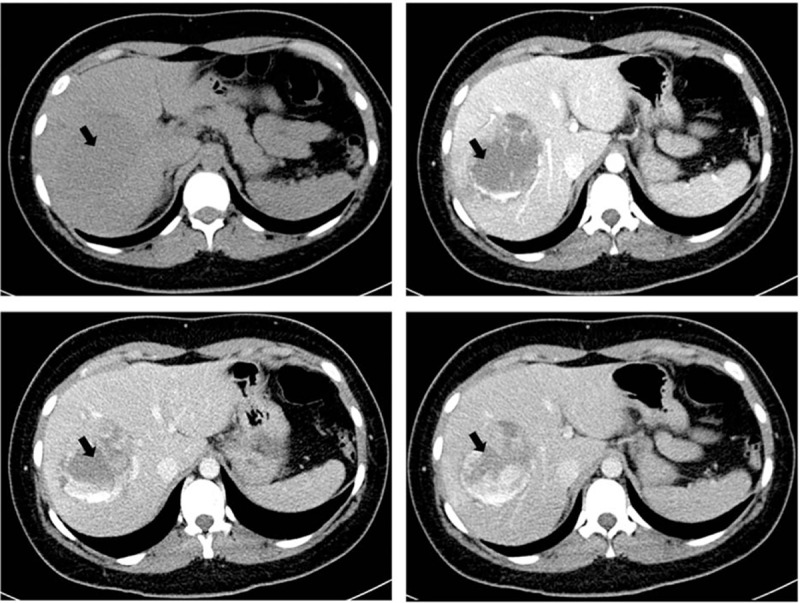
Unenhanced axial computed tomography (CT) scan and enhanced CT scan at arterial, portal, and later phases. Enhanced axial CT showing early leak of contrast material at the arterial phase due to arterial bleeding with increasing leak at the portal and later phases.

On clinical examination, her abdomen was tender but not tense, she was afebrile and hemodynamically stable. There was no history of trauma. Her laboratory tests showed: hemoglobin 13.5 g/dL, hematocrit 40%, liver enzymes: aspartate transaminase 196 U/L, alanine transaminase 239 U/L, bilirubin 14 μm/L, International Normalized Ratio 8.4, activated cephalin time of 180 seconds (ratio 5.4), a platelets count of 234,000 mm^−3^, a white blood cells count of 17,000 mm^−3^, a C-reactive protein of 19 mg/L. Abdominal ultrasonography revealed an unexpected large liquid picture measuring 10 cm by 5 cm over hepatic segments number VI, VII, and VIII. Abdominal computed tomography (CT) confirmed this suspicion of hematoma (Fig. 1).

**Figure 2:**
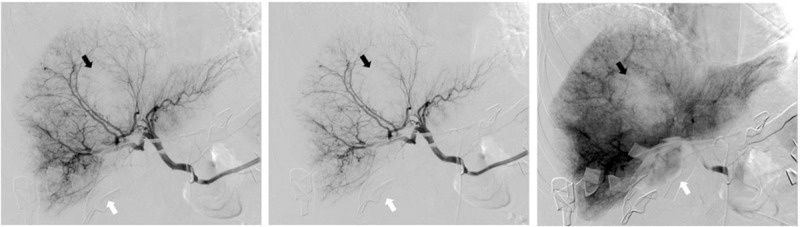
Frontal views of hepatic artery angiography after surgical packing (white arrow) showing no arterial bleeding or tumoral blush but a mass effect performed by intrahepatic hematoma (black arrow) and subcapsular hematoma.

A few hours later, she exhibits tachycardia, increased abdominal pain, and hypotension. Hemoglobin decreased to 5 g/dL. Fluid replacement therapy with crystalloids was started. Packed red blood cells and fresh frozen plasma transfusion were given. At the same time, fibrinogen concentrate (3 g) was administered (Clottafact [1.5 g/100 mL] LFB Biomedicaments, France). Decision was made to transfer the patient to a tertiary referring university hospital for endovascular embolization. The surgeon decided to implement abdominal packing as damage control therapy, to ensure safe conditions before transferring the patient. Active bleeding continued during the intervention and norepinephrine was introduced. At that time, 10 units of blood suspensions, 6 units of fresh frozen plasma, 4.5 g of fibrinogen concentrates, and 2 g of tranexamic acid were given. Selective catheterization of the 2 branches of the right hepatic artery was successfully performed (Fig. 2).

**Figure 3:**
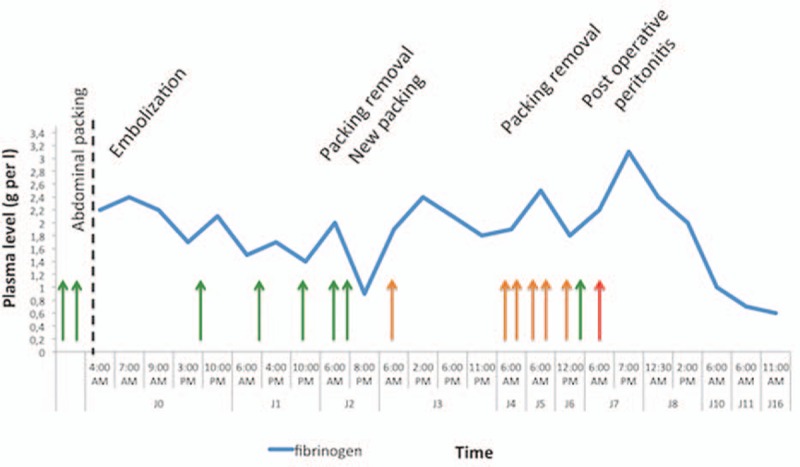
Fibrinogen activity during hospitalization in intensive care unit (ICU). Arrows: fibrinogen concentrates administration. (Green arrows: loading dose of 1.5 g; orange arrows: loading dose of 3 g, and red arrows: loading dose of 4.5 g).

On day 2, the patient underwent further surgery to remove abdominal packing but subcapsular hepatic hematoma was still bleeding and another packing therapy was necessary. Fibrinogen concentrates were infused just before this 2nd surgery to optimize coagulation disorders. The removal of abdominal packing was possible on day 4, without any abdominal bleeding (plasmatic fibrinogen 1.9 g/L before surgery, Von Clauss coagulation micromethod^[8]^). Three grams of fibrinogen concentrates and tranexamic acid were administered at the beginning of the 3rd surgery. The time course of plasma fibrinogen concentrations is described in Fig. 3. Simultaneously, she presented an early ventilator acquired pneumonia (methicillin sensitive *Staphylococcus aureus*). Despite an appropriate intravenous antimicrobial use, fever and ileus remained and white blood cells increased to 35,000 mm^−3^. On day 6, abdominal CT scan showed abundant pelvic peritoneal effusion and diffuse infiltration of the peritoneum, suggesting a postoperative peritonitis. Laparotomy was performed with abundant cleaning of the peritoneal cavity and drainage (plasmatic fibrinogen was 3.1 g/L after infusion of 4.5 g of fibrinogen concentrates). Antimicrobial therapy was switched to imipenem, vancomycin, and amikacin. No septic shock occurred. Purulent peritoneal effusion was infected with methicillin resistant *Staphylococcus haemolyticus* allowing de-scalation to vancomycin alone for 10 days. In the postoperative period, no bleeding occurred, bowel transit was normal, and drainage tubes were removed quickly. No thrombo-embolic adverse event occurred. Thrombo-embolic prophylaxis by low molecular weight heparin was introduced on day 7, after prophylactic intermittent legs compression during the 1st week. The patient was discharged to surgical ward on day 11.

## 3 Discussion

To the best of our acknowledge, this is the 1st case of spontaneous intrahepatic bleeding reported in patient with congenital afibrinogenemia whereas several cases of splenic ruptures were reported. One case of perioperative management of liver transplantation after Budd Chiari syndrome in a patient with afibriniogenemia was also reported.^[4,9]^ This case highlights the issues in the management of such severe bleeding complicated with infectious peritonitis related to abdominal repeated packing surgeries. Severe coagulopathy and inflammation are involved.

Severe hemorrhages in patients with afibrinogenemia can be treated with fibrinogen concentrates, cryoprecipitate, and fresh frozen plasma, but fibrinogen concentrates are the main option.^[2]^ They are virally inactivated and can be infused with small volumes and low allergy risk.^[10]^ In emergent conditions, fresh frozen plasma and cryoprecipitate are infused when fibrinogen concentrates are unavailable. The local hospital where the young women was 1st admitted did not assesse fibrinogen activity but we can hypothesize that it was lower than 0.9 g/L as reported by Peyvandi et al^[11]^ in European Network of Rare Bleeding Disorders. Prothrombin time and activated cephalin time were significantly prolonged as it is usually described in afibrinogenemia. United Kingdom Haemophilia Centre Doctors’ Organization recently published guidelines for the management of the rare coagulation disorders. For severe bleeding or major surgery in afibrinogenemia, fibrinogen concentrate 50 to 100 mg/kg, with smaller doses repeated if necessary at 2 to 4 day intervals to maintain fibrinogen activity >1.0 g/L, is recommended (2C).^[2]^ In this case of several packing surgeries, it was necessary to repeat fibrinogen concentrates administration and fibrinogen activity was always upper than 1 g/L. Nevertheless, hepatic bleeding continued until day 4. Previous reports found a 30% occurrence of venous or arterial thrombosis, nevertheless, no thrombo-embolic events occurred in this case despite high-dose fibrinogen replacement therapy associated with tranexamic acid infusion.^[12]^

The etiology of intrahepatic hemorrhage is usually secondary to trauma. Among the causes of spontaneous intrahepatic bleeding, the most common are hepatocellular carcinoma and hepatic adenoma. The diagnosis of hepatocellular carcinoma has been discarded on CT images. The hypothesis of hepatic adenoma was initially preferred in the context of a young woman with estrogen–progestin contraception and no history of liver cirrhosis.^[13]^ However, the diagnosis needs confirmation via a liver magnetic resonance imaging.^[14]^

The management of the intrahepatic hemorrhage is based on surgical techniques.^[15]^ Since the progress of interventional radiology procedures and advances in resuscitation, emergency surgery is now limited to failure of embolization procedure or absence of alternative therapy.^[16]^ In this case, management with radiological embolization was possible in 2nd-line due to the unavailability of arteriography in the local hospital. Given the hemodynamic instability, surgical treatment was necessary using packing by applying the principles of damage control surgery. This management allowed stabilization of the patient before transfer to the tertiary hospital.

Finally, fibrinogen plays a vital role in the process of inflammation, which is primarily mediated by its interaction with leucocytes through the surface receptors. It is also a ligand for intercellular adhesion molecule-1 and is an important mediator of cell–cell interaction and adhesion.^[17]^ In this case of secondary postoperative peritonitis and inflammatory process related to severe hemorrhage, we might hypothesize that needs for fibrinogen to ensure safe wound healing were probably much higher than in isolated nonsevere bleeding, the more so as a recent experimental animal study demonstrated that fibrin polymer formation is vital to *S aureus* clearance and ultimately host survival in *S aureus* induced peritonitis.^[18]^

## 4 Conclusions

In conclusion, spontaneous hepatic hematoma can be observed in patients with afibrinogenemia and is a real challenge for clinicians. Such as splenic rupture, it should be considered in patients with abdominal pain, acute abdomen, and hypotension or shock. Supportive treatment, damage control surgery with packing associated with selective embolization was successfully performed.
